# A Generic Procedure for the Isolation of pH- and Magnesium-Responsive Chicken scFvs for Downstream Purification of Human Antibodies

**DOI:** 10.3389/fbioe.2020.00688

**Published:** 2020-06-23

**Authors:** Steffen C. Hinz, Adrian Elter, Oliver Rammo, Achim Schwämmle, Ataurehman Ali, Stefan Zielonka, Thomas Herget, Harald Kolmar

**Affiliations:** ^1^Institute for Organic Chemistry and Biochemistry, Technische Universität Darmstadt, Darmstadt, Germany; ^2^Merck Lab @ Technische Universität Darmstadt, Darmstadt, Germany; ^3^Life Science Division, Merck KGaA, Darmstadt, Germany; ^4^Protein Engineering and Antibody Technologies, Merck KGaA, Darmstadt, Germany; ^5^Strategy und Transformation, Merck KGaA, Darmstadt, Germany

**Keywords:** affinity chromatography, yeast display, protein purification, immune library, chicken antibody, single chain fragment variable, downstream processing, protein A

## Abstract

Affinity chromatography provides an excellent platform for protein purification, which is a key step in the large scale downstream processing of therapeutic monoclonal antibodies (Mabs). Protein A chromatography constitutes the gold standard for Mab purification. However, the required acidic conditions (2.8–3.5) for elution from the affinity matrix limit their applicability, particularly for next generation antibodies and antibody fusion proteins, since denaturation and irreversible aggregation can occur due to the acidic buffer conditions. Here we describe a generic procedure for the generation of antigen-specific chromatography ligands with tailor-made elution conditions. To this end, we generated a scFv-library based on mRNA from a chicken immunized with human Fc. The antibody repertoire was displayed on yeast *Saccharomyces cerevisiae* screened *via* FACS toward pH- and magnesium-responsive scFvs which specifically recognize human IgG antibodies. Isolated scFvs were reformatted, produced in *Escherichia coli* and immobilized on NHS-agarose columns. Several scFvs were identified that mediated antibody binding at neutral pH and antibody recovery at pH values of 4.5 and higher or even at neutral pH upon MgCl_2_ exposure. The iterative screening methodology established here is generally amenable to the straightforward isolation of stimulus-responsive antibodies that may become valuable tools for a variety of applications.

## Introduction

In the time period of 2014 to 2018 a total of 129 unique biopharmaceuticals were approved for either the US or EU market raising the total number of approved biopharmaceuticals to 316. The category of biopharmaceuticals contains monoclonal antibodies (mAbs), hormones, clotting factors, enzymes and others. This heterogeneous group of molecules is mainly utilized in cancer, inflammation-related, hemophilia and diabetes indications and is responsible for $188 bn sales in 2017 with expectations to reach nearly $400 bn in 2025 (Grand View Research, 2017). With up to 65.5% of total sales, monoclonal antibodies remain the most interesting protein scaffold. In the recent decade, the market became more congested and multiple molecules are utilized for similar indications. This leads to an increase in competitive pressure, a motivation for research and development of new low-cost production and purification strategies (Walsh, 2018).

In the production process of proteins, downstream processing is one of the major cost driving factors, accountable for 45–90% of the whole manufacturing process costs, making it a promising target for optimization (Straathof, 2011). Affinity chromatography is a powerful tool for efficient purification of the protein of interest (POI) omitting the need for multiple chromatography steps (Harakas, 1994; MacLennan, 1995; Saraswat et al., 2013; Urh et al., 2009). However, the genetic fusion of affinity tags to the POI is a putative origin for immune reactions which should be avoided (Dingman and Balu-Iyer, 2019). In the case of antibodies, natural occurring affinity ligands exist, omitting the need for affinity tags. These bacterial proteins can be found in *Staphylococcus* species (Protein A, Protein G) or *Peptostreptococcus* species (Protein L) and mask the bacteria from the immune system of the host organism and are also known as virulence factors (Ricci et al., 2001; Palmqvist et al., 2002). This strong natural interaction can be exploited for affinity chromatography, where antibodies can be efficiently bound onto a Protein A-agarose column (Duhamel et al., 1979). This natural affinity comes with a drawback. For the interruption of this tight interaction, harsh elution conditions have to be applied. Commonly used Protein A chromatography relies on glycine/citrate buffer compositions with a pH of 2.8–3.5 to achieve high recovery. These acidic conditions are not well tolerated by some antibodies and may lead to protein loss by aggregation (Vázquez-Rey and Lang, 2011; Mazzer et al., 2015; Jin et al., 2019) as well as structural changes such as deamidation and backbone cleavage upon succinimide formation (Linhult et al., 2005; Lu et al., 2019) and therefore result in less economical production conditions. Intensive efforts have been made to improve Protein A/G to overcome this intrinsic drawback. On one hand, the high costs of Protein A/G columns have been countered by improvements toward alkaline stability, allowing more purifications cycles per column (Nilsson et al., 1987; Gulich et al., 2002; Hahn et al., 2006). On the other hand, efforts have been made to establish less acidic elution conditions by Protein A/G engineering and rational design culminating in variants that can be used with elution at pH 4.5 (Gulich et al., 2000; Watanabe et al., 2009, 2019; Pabst et al., 2014; Tsukamoto et al., 2014). Alternative approaches focus on temperature-dependent elution (Koguma et al., 2013) or antibody binding upon calcium supplementation and subsequent elution with EDTA at neutral pH (Kanje et al., 2018; Scheffel et al., 2019).

Several alternative binding proteins have been developed for Fc affinity purification purposes. For example, single-domain antibody domains (VHH) have been isolated from immunized camelids. For their use in downstream processing applications gentle elution conditions should prevail and these antibodies were therefore engineered toward pH- and magnesium-responsive binding behavior (Klooster et al., 2007; Detmers et al., 2010; Hermans et al., 2014). Further developments focus on semi-synthetic protein scaffold libraries based on i.e., a DNA-binding protein derived from a hyperthermophilic crenarchaea called Sso7D (“Affitins”) (Gera et al., 2012; Behar et al., 2016), “Affimers” based on cystatin and human stefin A (McPherson and Tomlinson, 2014; Tiede et al., 2017), modular leucine-rich repeat units called “Repebodies” (Heu et al., 2014), and many more as elegantly reviewed by Škrlec et al. (2015) not only limited to affinity chromatography but also for imaging purposes and therapeutic applications (Bedford et al., 2017; Simeon and Chen, 2018).

The goal of this study was to establish a generic screening procedure for pH- and/or magnesium-responsive binders against a given target *via* yeast surface display of a single chain fragment variable (scFv) library obtained from immunized chickens for use in affinity chromatography applications. Here we describe a screening strategy that allows for isolation of high affinity target binding antibodies that release their target at slightly acidic pH or in combination with pH drop and elevated magnesium concentrations. We used chickens for immunization since the phylogenetic distance between avians and humans is significantly increased, enhancing the probability to obtain antibodies against human antigens bypassing antigen self-tolerance related problems (Davies et al., 1995). Also, chicken antibodies tend to be structural more rigid caused by non-canonical disulfide bridges located in the CDRs and elevated body temperature of avians (Prinzinger et al., 1991; Wu et al., 2012). Additionally, the special genetic arrangement of the VH and VL gene of chickens allows the amplification of the whole antibody repertoire with two separate one-step PCRs using defined primer pairs (Reynaud et al., 1985, 1987, 1989, 1991; Thompson and Neiman, 1987; McCormack et al., 1993).

Here we show that the combination of avian immunization, yeast surface display (YSD) and ultra-high throughput screening by FACS results in the isolation of numerous human Fc-specific pH- and magnesium-responsive scFvs. After immobilization they displayed favorable binding and elution profiles and allowed for target antibody purification using mild elution conditions.

## Materials and Methods

### Immunization

The immunization of a pathogen-free adult laying hen (*Gallus gallus domesticus*) was performed at Davids Biotechnologie GmbH, Germany. The specimen was intramuscularly injected with 150 μg SEED-Fc-protein (gift from Dr. S. Zielonka, Merck KGaA, Darmstadt) (Davis et al., 2010; Muda et al., 2011) in combination with immune adjuvant AddaVax (InvivoGen). Boosters were given 2, 4, 5, and 8 weeks after the initial injection. Immune response against the Fc-protein was determined after 5 weeks by antigen-specific titer determination using ELISA. The chicken was sacrificed after 9 weeks and the total splenic RNA was subsequently isolated using TriFast reagent (VWR).

Experimental procedures and animal care were in accordance with EU animal welfare protection laws and regulations.

### Yeast Strains and Media

*Saccharomyces cerevisiae* EBY100 [*MATa URA3-52 trp1 leu2Δ1 his3Δ200 pep4::HIS3 prb1Δ1.6R can1 GAL (pIU211:URA3*)] (Thermo Fisher Scientific) cells were utilized for yeast surface display. Prior to library generation, yeast cells were cultivated in YPD medium composed of 20 g/L peptone/casein, 20 g/L glucose and 10 g/L yeast extract supplemented with appropriate antibiotics (ampicillin 100 mg/L, kanamycin sulfate 75 mg/L or chloramphenicol 25 mg/L). The SD-CAA media for yeast cell cultivation after library generation comprised 5.4 g/L Na_2_HPO_4_ and 8.6 g/L NaH_2_PO_4_ × H_2_O, 20 g/L glucose, 5 g/L ammonium sulfate, 1.7 g/L yeast nitrogen base (without amino acids), and 5 g/L bacto casamino acids. For surface presentation induction, SG-CAA medium was used which resembles SD-CAA but instead of glucose, galactose was supplemented.

### Library Construction

The generation of the scFv gene library was performed as described by Grzeschik et al. (2019). Briefly, the VH and VL genes were amplified with *VH_gr_up*/*VH_SOE_lo* and *VL_SOE_up*/*VL_gr_lo*, respectively (Supplementary Table S3), after cDNA synthesis and subsequent mRNA digestion. Both fragments were fused in two additional PCR reactions introducing a (Gly_4_Ser)_3_ linker sequence, amplifying the scFv gene while adding overhangs at the 5′ and 3′ end of the scFv gene for homologous recombination in yeast. The PCR amplicon was subsequently purified. The pCT plasmid (Boder and Wittrup, 1997) which was used for surface presentation was digested with *Nhe*I and *BamH*I (New England Biolabs) and purified prior to gap repair cloning. The library generation with scFv insert, pCT backbone and EBY100 yeast cells were performed according to Benatuil et al. (2010).

Twelve transformation reactions were performed each consisting of 4 μg digested pCT plasmid and 12 μg scFv insert. The number of individual transformants was determined by dilution plating after 3 days of cultivation at 30°C.

### Library Sorting

Prior to yeast cell sorting, the yeast library was cultivated overnight in SD-CAA medium at 30°C and 180 rpm. Subsequently, the cell suspension was centrifuged at 4000 rcf and the medium aspirated. The cell pellet was utilized to inoculate SG-CAA medium to a final cell density of 1 × 10^7^ cells/mL followed by an incubation period of at least 12 h at 30°C. Cells were harvested by centrifugation and washed once in 1 mL PBS-B pH 7.4 [PBS + 0.1% (w/v) BSA] prior to the staining procedure.

The presentation of the scFv molecules on the yeast cell surface was verified either with an anti-c-myc antibody (produced in house) and anti-mouse IgG FITC (Sigma Aldrich) or anti-mouse IgG R-phycoerythrin (PE) (Sigma Aldrich) as secondary labeling agent or anti-c-myc biotin antibody (Miltenyi biotech) and Streptavidin-PE (Fisher Scientific) or Streptavidin-allophycocyanin (APC) (Fisher Scientific), respectively. The detailed staining strategy including working dilution is summarized in Supplementary Table S1. The staining steps are summarized in Supplementary Table S2.

**FIGURE 1:**
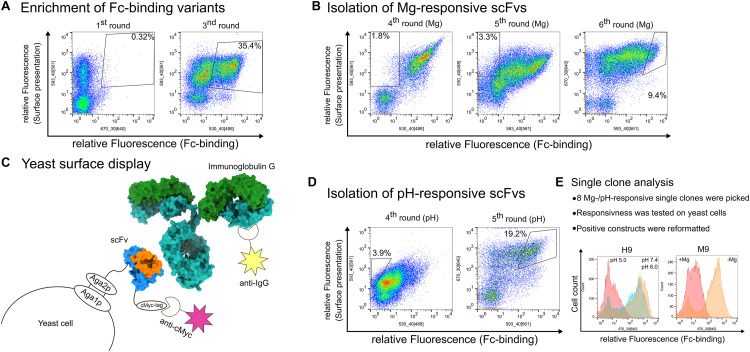
Summary of the sorting steps for the isolation of pH- and magnesium-responsive chicken-scFvs. In general, FACS plots show Fc-binding plotted on the *x*-axis and surface presentation on the *y*-axis. Each plot contains 50,000 events. Detailed staining strategy is summarized in Supplementary Table S1. **(A)** Enrichment of Fc-specific scFvs *via* YSD and FACS during the 1st and 2nd sorting rounds. **(B)** Summarized screening campaign for the isolation of magnesium-responsive scFvs. The 3rd and 4th sorting rounds were performed to deplete scFv variants that bound regardless of the MgCl_2_ concentration by sorting non-binding yeast cells after incubating the cells with 2 M MgCl_2_. The 5th sorting round was conducted in order to enrich IgG binding cells and to isolate single clones. **(C)** Schematic depiction of yeast surface display. The scFv molecule (pdb: 4p48) is displayed on the yeast cell *via* aga2p-fusion. Surface presentation is verified by staining the *C*-terminal c-Myc-tag. The scFv backbone is shown in blue, the CDRs are highlighted in orange. Antibody binding (pdb: 1igt) is verified indirectly by fluorophore staining. The heavy chains are depicted in light blue, the light chains in green. **(D)** Consecutive sorting rounds to enrich pH-responsive scFvs. The 3rd sorting round was conducted with an additional incubation step at pH 5.0 and non-binding yeast cells were isolated. The 4th sorting round was performed in order to isolate yeast single clones. **(E)** General procedure for the analysis of single clones. Exemplarily, H9 and M9 analysis is depicted in a histogram, respectively. Fc-binding is depicted on the *x*-axis and cell count on the *y*-axis. For H9, cells washed at pH 7.4 after target protein incubation are shown in orange, cells washed at pH 6.0 in blue and cells washed at pH 5.0 are shown in red. M9-bearing cells are shown in the red histogram with non-Magnesium exposed cells are shown in orange and cells exposed to 2 M MgCl_2_ are shown in red.

In the screening process, daratumumab (Janssen-Cilag), pertuzumab (Genentech) and cetuximab (Merck) were utilized. Antibody binding was detected with anti-Human IgG (Fab specific) -FITC (Sigma-Aldrich), Goat F(ab’)_2_ anti-Human Kappa-PE (Southern Biotech) or Goat anti-Human IgG Fc Secondary Antibody PE (Invitrogen) (Figure 1C). In the first and second screening round SEED Fc protein (Merck KGaA) was utilized conjugated with DyLight650 (Thermo Fisher Scientific). Conjugation was performed according to the manufacturer’s manual with 5-fold molar excess of NHS-DyLight650.

Yeast cells that were collected during the sorting process were transferred into SD-CAA medium and cultivated for 48 h at 30°C and 180 rpm before re-activating surface presentation by inoculating SG-CAA medium for subsequent sorting. In each sorting round, the total number of screened cells exceeded 10-fold the number of cells in the previous sorting process to ensure proper library coverage during sorting.

### Bacterial Strains and Media

*Escherichia coli* Top10 cells [F^–^ *mcr*A Δ(*mrr*-*hsd*RMS-*mcr*BC) φ80*lac*ZΔM15 Δ*lac*X 74 *rec*A1 *ara*D139 Δ(*ara*-*leu*) 7697 *gal*U *gal*K *rps*L (Str^R^) *end*A1 *nup*G λ-] (Thermo Fisher Scientific) were used for transformation of golden gate assembly batches. Cell cultivation was performed in dYT medium (yeast extract 10 g/L; peptone ex casein 16 g/L; NaCl 5 g/L). Correctly assembled plasmids were rescued and subsequently transformed into the SHuffle T7 Express strain (*fhuA2 lacZ::T7 gene1* [lon] *ompT ahpC Etc gal λatt:*:pNEB3-r1-*cDsbC* (Spec^R^, *lacI*^q^) Δ*trxB sulA11 R(mcr-73::miniTn10*–Tet^S^) [dcm] *R(zgb-210::Tn10* –Tet^S^) *endA1 Δgor Δ(mcrC-mrr)114::IS10*) (New England Biolabs) for expression. SHuffle T7 Express cells were cotransformed with the pLysS Erv1p/DsbC plasmid (Veggiani and de Marco, 2011) and transformants were maintained upon addition of 60 μg/ml kanamycin and 25 μg chloramphenicol. Expression in SHuffle T7 Express was conducted in SB medium (yeast extract 20 g/L; peptone ex casein 32 g/L; NaCl 5 g/L; NaOH 1 N 5 mL).

### Cloning, Expression, Purification of Solitaire scFvs

Single clones with pH-responsive or magnesium-responsive properties were reformatted into a pET30a expression plasmid comprising *Bsa*I-HF^®^v2 sites for golden gate cloning (GGC). To this end, scFv genes were amplified from the respective pCT plasmid using *scFv_chick_his_GG_up* and *scFv_chick_SII_GG_*lo primers (Supplementary Table S3) introducing *Bsa*I restriction sites for golden gate assembly as well as a 5′ His6-tag and 3′ StrepTagII (Johar and Talbert, 2017) for purification. The golden gate assembly was performed with 75 ng pET30a backbone and 25 ng of the purified PCR amplicon. Plasmids with the correct sequence were subsequently transformed into SHuffle T7 Express *E. coli* cells containing a pLysS Erv1p/DsbC plasmid.

The expression of the scFvs was performed according to Veggiani and de Marco (2011) in SB medium with a final concentration of 0.1% arabinose and 1 mM IPTG at 18°C, 180 rpm for at least 16 h.

Cells were harvested by centrifugation, lysed by sonication and the soluble fraction was applied onto a HisTrap HP 5 mL column (GE Healthcare) for purification. Chromatography was performed on an ÄKTA Start FPLC system. As binding buffer IMAC Buffer A (Tris/HCl 50 mM pH 7.5, NaCl 150 mM) was utilized. Bound protein was eluted with IMAC Buffer B (Tris/HCl 50 mM pH 7.5, NaCl 150 mM, 1 M imidazole) in a linear gradient. After the IMAC, the protein containing fractions were pooled and diluted 1:2 with Buffer W (Iba Lifescience). As a second purification step, the protein containing solution was applied on a StrepTactin XT high capacity column (Iba Lifescience). Column wash was performed with Buffer W and bound protein was eluted with Buffer E (Iba Lifescience). Buffer exchange was performed with a dialysis hose with a MWCO of 3 kDa with at least 2 dialysis steps.

### Protein Immobilization

Purified scFv protein was coupled to prepacked NHS-agarose columns (GE Healthcare, 1 ml column volume) according to the manufacturers protocol. To this end, 5 mg protein (in PBS buffer) were slowly applied to the column with a contact time of approx. 500 μl/15 min after rinsing the column with 6 ml ice-cold HCl (1 mM) *via* a syringe. The column was cleared from non-immobilized protein by washing the column with 2 ml PBS. Subsequently, the column was washed with 6 ml Buffer Q (500 mM Tris-HCl, 500 mM NaCl, pH 8.3), 6 ml Buffer QB (100 mM sodium acetate, 500 mM NaCl, pH 4.0) and 6 ml Buffer Q. After an incubation time of 60–90 min in Buffer Q to quench unreacted NHS-groups, the column was subsequently washed with 6 ml Buffer QB, 6 ml Buffer Q and 6 ml Buffer QB, respectively. In the last step, the column was purged with coupling buffer (PBS) and stored at 4°C until further utilization.

The coupling efficiency was determined utilizing the Pierce 660 nm Protein Assay Reagent. Briefly, the flowthrough of the coupling solution as well as the 3 ml coupling buffer wash solution were collected after passing through the column. Fifty μL of the solution was mixed with 750 μL assay solution. After 5 min incubation at RT, the absorbance at 660 nm was measured. For each protein, a unique calibration curve was recorded which was utilized to calculate the protein concentration in the flowthrough.

### Nano Differential Scanning Fluorimetry Measurement

Melting point measurements were performed on a Prometheus NT.48 instrument (NanoTemper Technologies). Ten μl of a 1 mg/ml protein solution in PBS was filled into the capillaries and a temperature gradient of 2°C/min was applied from 20 to 95°C. During the gradient, the fluorescence at 330 and 350 nm were measured to determine the melting temperature T_M_. Data processing was performed using the Prometheus ThermControl v.2.1.2.

### Downstream Processing Test Procedure

ScFv-conjugated columns were analyzed on a Bio-Rad NGC FPLC system. Each column was washed with sample buffer for at least 10 CV prior testing. The testing protocol included the following chromatography steps: (1) 5 CV sample buffer; (2) 5 CV elution buffer; (3) 5 CV sample buffer; (4) Sample application [3 mg Gamunex (Bayer)]. Gamunex is a therapeutic product for patients with an immunoglobulin deficiency. It contains a mixture of 98%-pure IgG-isotypes with a ratio of IgG1 62.8%, IgG2 29.7%, IgG3 4.8%, and IgG4 2.7%. (5) 10 CV sample buffer; (6) 5 CV sample buffer/elution buffer (mixed in variable ratios); (7) 5 CV elution buffer; (8) 5 CV sample buffer. Depending on the performed test series, phosphate citrate pH 7.4 was utilized as sample buffer (pH-test series) or Tris/HCl 50 mM pH 7.0 (magnesium-test series), respectively. As elution buffer, either phosphate citrate pH 3.0 or Tris/HCl 50 mM pH 7.0, MgCl_2_ 2 M was utilized. To evaluate the recovery at the utilized pH/magnesium concentration, the area under curve for the first elution step was divided by the total area under curve for both peaks multiplied by 100, resulting in percent values. Unless otherwise indicated, pH test series were performed at flowrates of 3 mL/min (except sample application with 1 mL/min) whereas magnesium test series were performed at 1 mL/min.

### Expression of Trastuzumab

For the purification experiments, Trastuzumab was produced according to published procedures (Schneider et al., 2019). The purification was performed either with a Protein A HP 1 mL column (GE Healthcare) or a self-produced column with 15 mL cell culture supernatant (diluted 1:2 with phosphate citrate buffer pH 7.4) with a two-step purification protocol described in the downstream processing test procedure paragraph.

### Biolayer Interferometry

The binding kinetics were determined utilizing the Octet RED96 system (ForteBio, Pall Life Science). Measurements were performed at 30°C and 1000 rpm. All biosensors were incubated in PBS prior to utilization for at least 10 min. After determination of the baseline in PBS buffer for 30 s, the anti-human Fab-sensors (FAB2G) were loaded with daratumumab at a concentration of 1 μg/ml in PBS for 300 s. Quenching of the biosensors was performed for 60 s in kinetic buffer (ForteBio, Pall Life Science) prior to incubation of the sensors with varying concentrations (25–800 nM) of scFv in PBS for 600 s (H9; M9) or 720 s (M2). The dissociation of the antigen was measured for 600 s in phosphate-citrate-buffer at the respective pH (pH 5.0, pH 4.5, or pH 4.0). For each BLI experiment, a negative control was measured in which the biosensor was incubated in PBS instead of the IgG1 antibody. This negative control was subtracted from all antigen containing samples. Data analysis was performed with ForteBio data analysis software 9.0 using a 1:1 binding model with Savitzky–Golay filtering. For *K*_D_ determination, at least three different scFv concentrations were used for a global fit. For the dissociation, only the fast dissociation step was considered for calculation (H9: 45 s; M2: 30 s; M9: 30 s) as described by Molecular Devices (Renee and Weilei, 2017). The *K*_dis_ values in different buffers were measured at single concentrations (H9: 500 nM; M2: 400 nM; M9: 250 nM).

## Results

Our previous immunization campaigns of chickens indicated that a high diversity of antibodies against mammalian antigens can be obtained (Grzeschik et al., 2019). Hence, we reasoned that this repertoire might contain several antibodies that display *per se* pH- and/or magnesium responsive binding. To test this hypothesis, we used a human SEED-Fc fragment as antigen for chicken immunization. A SEED-Fc consists of an IgG CH2 domain and a strand-exchanged IgG CH3. The strand exchanges were made with an CH3 of an IgA (Davies et al., 1995). A serum sample withdrawn 35 days after immunization indicated a strong immune response (data not shown). RNA was isolated from spleen tissue and cDNA was prepared by reverse transcription. The cDNA was amplified by PCR and VL und VH chains were randomly combined into scFv fragments by SOE PCR as described (Grzeschik et al., 2019). After transfer into yeast display vector pCT by gap repair (Benatuil et al., 2010) a yeast display library was generated in 1 day comprising approximately 5.2 × 10^9^ transformants.

To exclusively obtain Fc binding antibodies, four different antigens [daratumumab (Janssen-Cilag), pertuzumab (Genentech), cetuximab (Merck), Fc-fusion protein] all sharing a common human IgG1-Fc fragment, were used for screening. Starting with 1.6 × 10^8^ clones of the initial library which displayed a surface presentation level of 37.8% (Supplementary Figure S1), obviating the need for a magnetic bead preselection step, two consecutive FACS sorting rounds were performed with 1 μM Fc-fusion in the first sorting round and after cell growth with 1 μM of the human therapeutic antibody daratuzumab in the second sorting round to enrich Fc-binding scFv variants. To this end, yeast cells were sorted that showed fluorescence indicative of target binding and also for yeast surface presentation of the scFv resulting in 0.32% (round 1) and 35.4% (round 3) sorted yeast cells (Figure 1A). After the second sorting round, strong enrichment of binders was observed (Figure 1A). Afterward, the library was split into two different batches. One batch was further utilized to isolate pH-responsive scFvs whereas the second batch was utilized to screen for Mg-responsive scFvs (from now on called pH-batch and Mg-batch, respectively). In order to isolate pH-responsive scFvs, the yeast cells from the pH-batch were stained with 250 nM pertuzumab and FITC-labeled anti-Fab antibody. Afterward, cells were incubated at pH 5.0 for 5 min and those yeast cells were isolated that showed surface presentation but lacked target binding (Figure 1D). Prior to single clone analysis, a 5th sorting round was performed using 150 nM daratumumab to isolate single clones that showed target binding at pH 7.4 (Figure 1D). All FACS plots showing the sorting for pH-responsive scFvs are depicted in Supplementary Figure S2.

Magnesium-responsive scFvs were generated within three consecutive FACS rounds after the initial library enrichment. To this end, two consecutive selection rounds were performed by screening for non-binders upon indirect cell staining with 1 μM cetuximab followed by subsequent incubation in Tris/HCl buffer pH 7.0 with 2 M MgCl_2_. Sorted cells lacking the target binding fluorescence signal under these conditions increased from 1.8 to 3.3%, respectively (Figure 1B). The last sorting round prior to single clone analysis was performed *via* screening for binders of 150 nM daratumumab with no MgCl_2_ added to ensure enrichment of Fc-specific binders after two rounds of sorting for non-binding scFvs. The complete sorting campaign is shown in Supplementary Figure S3.

Eight pH- and nine magnesium-responsive single clones were picked and analyzed toward their binding properties in either phosphate-citrate pH 5.0 buffer (Dawson et al., 1987) or Tris/HCl 2 M MgCl_2_ buffer (pH 7.0), respectively. To this end, 5 × 10^6^ yeast cells of each putative pH-responsive single clone were collected by centrifugation and stained with 100 nM pertuzumab and APC labeled anti-kappa antibody for target binding and a biotinylated anti-myc-epitope antibody followed by streptavidin R-phycoerythrin (SPE) staining for surface presentation (Supplementary Figure S4A). Except for single clone H2, all variants showed pH-responsive behavior and were therefore considered for reformatting and expression. In FACS analysis, H7 and H9 exhibited significant loss of target binding at pH 5.0 (exemplarily shown for H9 in Figure 1E). Putative yeast-displayed magnesium responsive variants were tested toward target binding *via* FACS with 150 nM daratumumab (exemplarily shown for M9 in Figure 1E, data for all single clones depicted in Supplementary Figure S4B). Single clone 1 and 7 showed no or only minor target binding decrease after MgCl_2_ treatment and were therefore excluded from further experiments. Responsive variants were sequenced and reformatted into a pET30 expression plasmid *via* golden gate cloning with a *N*-terminal His6-tag and a *C*-terminal StrepTagII to be used as purification handles. Seven unique pH-responsive variants were expressed which could be allocated to four different sequence clusters (H1–H9). Likewise, the magnesium-responsive variants showed high diversity with five unique variants out of eight belonging to four different clusters (M2-M9) (Table 1).

**TABLE 1:** Summary of TM values at pH 7.4 and pH 3.0 for all isolated pH- and magnesium-responsive scFvs including the observed yields and determined coupling efficiencies.

	Cluster	T_M_1 pH 7.4	T_M_2 pH 7.4	T_M_1 pH 3.0	T_M_2 pH 3.0	Yield [mg/L]	Coupling [%]
H1	III	60.5	68.4	40.55	50.00	4.7	n.d.
H3	II	45.7*	–	n.d.	–	5.2	–
H4	I	55.4	–	27.8	–	12.7	96.5
H6	I	58.5	–	43.8	–	16.9	71.1
H7	I	57.95	70.35	43.85	56.5	4.8	n.d.
H8	II	52.3	–	49.0	–	24.4	71.3
H9	I	48.7	55.8	33.3	42.15	5.38	n.d.
M2	I	51.0	–	32.5	–	10.9	78.8
M3	II	61.2	–	38.1	–	8.1	67.2
M6	III	53.55	–	35.3	–	3.6	n.d.
M8	III	52.45	–	n.d.	–	5.14	75.1
M9	III	69.48	–	59.4	65.5	14.8	80.6

The melting temperature for every expressed protein was determined by Nano differential scanning fluorimetry. The measurements showed T_M_ values in line with published melting temperatures for chicken scFvs ranging from 48 and 68°C (Table 1) with H1, M3, M9 having melting temperatures exceeding 60°C, comparable to Fab molecules (Orr et al., 2003; Garber and Demarest, 2007; Miller et al., 2010). For some variants, two distinct melting temperatures could be observed. This might be due to different melting temperatures for the VL and the VH domain (Miller et al., 2010).

For the chromatography experiments, 5 mg of each scFv were utilized for coupling to NHS-agarose columns. The coupling efficiency varied between 67 and 96% (Table 1). Prepared columns were stored at 4°C until further utilization. All chromatographic procedures were performed on a NGS Bio-Rad system with pH-module calibration between each column experiment set. A column experiment set includes a pH-gradient (pH 3.0–7.4) and up to eight different isocratic elution chromatographies for pH-experiments or a MgCl_2_-gradient (0–2 M MgCl_2_) and up to five different isocratic elution chromatographies, respectively.

**FIGURE 2:**
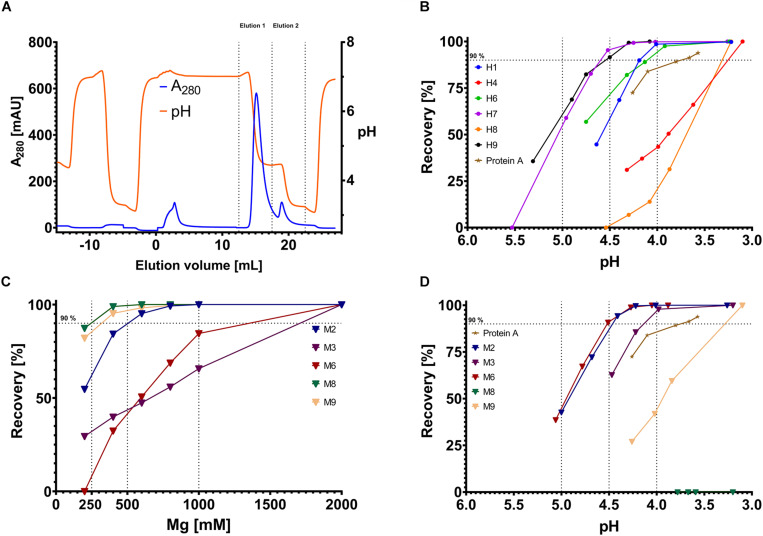
Example chromatogram and recovery diagrams. **(A)** Sample chromatogram with the absorbance at 280 nm depicted on the *y*-axis (blue line) and the elution volume on the *x*-axis. Additionally, the recorded pH is shown on the right *y*-axis in orange. Elution 1 and elution 2, which are used for determining the recovery, are indicated by dashed lines on the *x*-axis. **(B,D)** Recovery for pH-responsive scFvs **(B)** and magnesium-responsive scFvs. The recovery is depicted on the *y*-axis, the pH value on the *x*-axis. Each dot represents a single measurement (triangles for magnesium-responsive variants), scFvs are separated by color, Protein A data points are depicted as stars. Vertical dashed lines indicate pH 4.0, pH 4.5 and pH 5.0, respectively, horizontal dashed lines indicate 90% recovery. **(C)** Depiction of the recovery of Magnesium-responsive chicken scFvs. Dashed vertical lines indicate 250 mM, 500 mM, or 1000 mM MgCl_2_ concentration.

To determine the recovery, a two-step isocratic elution was performed. The first isocratic step was performed in phosphate-citrate-buffer with variable pH (Figure 2A – Elution 1). The second elution step was performed in phosphate-citrate-buffer pH 3.1–3.3 ensuring the elution of remaining IgG on the column (Figure 2A – Elution 2). The area under curve (AUC) was determined for each elution step. The AUC ratio of the first elution step and the sum of the first and second elution step resulted in a percentage value which defines the recovery. In comparison with Protein A, all proteins except H4 and H8 showed higher recovery, exceeding 90% at pH values >4.0. The best variants, H7 and H9, displayed recovery of more than 92% above pH 4.5 (Figure 2B).

The pH-experiments were also conducted with the magnesium-responsive variants. Interestingly, three out of five scFvs displayed pH-responsive behavior (Figure 2C). Particularly M2, M6, and M3 revealed pH- responsive elution behavior even outperforming Protein A at pH 4.0. M8 did not show any elution at pH 3.2.

**FIGURE 3:**
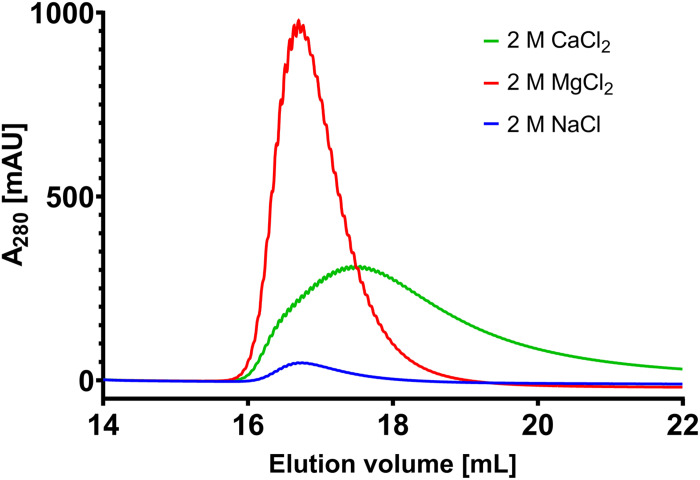
Selectivity test of M9 toward CaCl_2_, NaCl and MgCl_2_. Depiction of the elution of 2 mg Gamunex bound to immobilized M9 by 2 M NaCl (blue), 2 M CaCl_2_ (green) or 2 M MgCl_2_ (red), respectively. Recovery as determined by the AUC was 98.8% for MgCl_2_, 79.3% for CaCl_2_ and 8.2% for NaCl, respectively.

In the chromatography experiments with MgCl_2_ as eluant, M8 was the best performing scFv showing quantitative elution at 400 mM MgCl_2_, closely followed by M9. M2 performed adequately with nearly quantitative elution at 800 mM MgCl_2_. M3 and M6 only showed mediocre magnesium-responsiveness with only 55 and 65% elution at 1000 mM MgCl_2_, respectively (Figure 2D). As negative control, H7 and H9 were tested toward their magnesium-responsiveness but did not show any protein elution signal at 2 M MgCl_2_. To see whether the elution was driven by high ionic strength or specifically by Mg^2+^ ions, bound antibody on scFv M9 column was incubated with Tris/HCl buffer pH 7.0 containing either 2 M NaCl, 2 M CaCl_2_ or 2 MgCl_2_, respectively. It was apparent, that antibody elution was specific for bivalent cations, with strong preference for Mg^2+^. In direct comparison, Ca^2+^ only lead to a recovery of 80% with very strong tailing, clearly demonstrating Mg^2+^ specificity (Figure 3).

**FIGURE 4:**
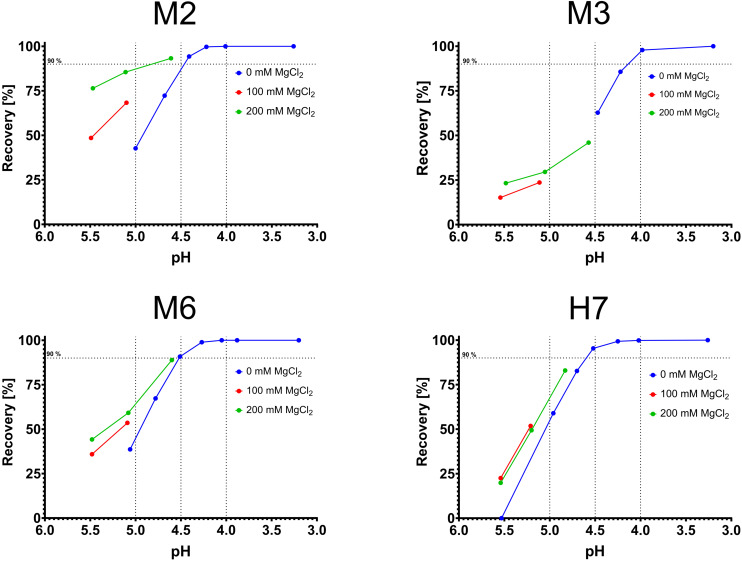
Combinatorial experiments with varying magnesium concentration and varying pH values. pH is depicted on the *x*-axis, recovery on the *y*-axis. Blue data points were ascertained in phosphate buffer (0 M MgCl_2_), red data points in phosphate buffer with 100 mM MgCl_2_ and green data points in phosphate buffer with 200 mM MgCl_2_.

An unexpected finding was the pH-dependent elution of M2, M3, and M6. All three variants were examined in combinatorial experiments with phosphate-citrate-buffer (pH 5.5–4.5) and low concentrations of MgCl_2_ (0, 100, 200 mM). As expected, the addition of MgCl_2_ significantly improved the recovery for all tested variants (Figures 4A–C). As negative control, H7 was also tested toward the combinatorial effects of MgCl_2_ and pH. The addition of 100 mM MgCl_2_ was beneficial at pH 5.5 and 5.2 and slightly improved recovery. However, further increasing the MgCl_2_ concentration did not further improve the elution behavior (Figure 4D).

**FIGURE 5:**
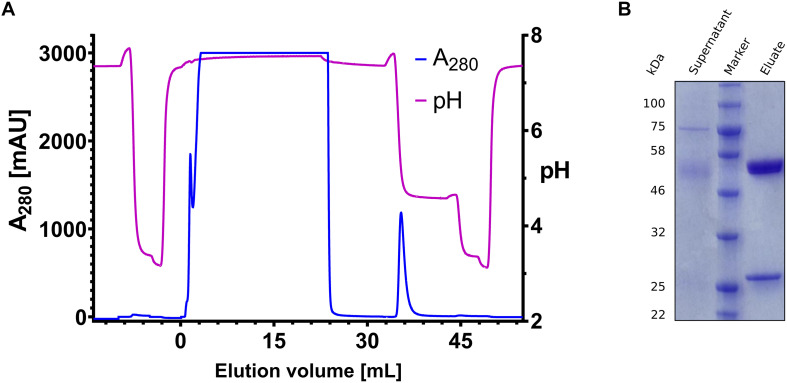
Trastuzumab purification from trastuzumab producing Expi293F cell culture supernatant. **(A)** Chromatogram of the purification of trastuzumab from 15 mL Expi293 supernatant. Absorbance at 280 nm is depicted on the left *y*-axis (blue), pH is shown on the right *y*-axis (violet). The elution volume is depicted on the *x*-axis. Elution was performed in two steps with pH 4.6 and pH 3.0, respectively. **(B)** SDS-PAGE gel with the supernatant on the left lane and the purified sample on the right lane, the protein ladder (Blue Prestained Protein Standard, Broad Range; New England Biolabs) is depicted on the middle lane.

The most pH-responsive affinity ligands H7 and H9 were utilized to investigate, whether the recovery can be further enhanced by lowering the flow rate during elution from 3 ml/min to 1 ml/min. It could be shown, that decreasing flow rate gains 3–5% recovery (Supplementary Figure S5). In order to evaluate the specificity of H9, an Expi293F culture was transiently transfected with expression plasmids coding for trastuzumab heavy chain and trastuzumab light chain, respectively. After 5 days of expression, the supernatant was filtered, diluted 1:2 with phosphate citrate buffer pH 7.4 and directly applied onto the column. The elution was performed with phosphate citrate buffer at pH 4.6 in the first elution step. As revealed by SDS-PAGE, it was possible to isolate trastuzumab from mammalian cell culture supernatant at high yield and purity with a total recovery of 98.7% (Figure 5). A likewise performed purification with a Protein A HP 1 ml column showed no elution at pH 4.6 and the antibody eluted at pH 3.0 off the column (Supplementary Figure S9). Both purification procedures showed comparable elution profiles, resulting in antibody recovery either at pH 4.6 (H9 functionalized column) or pH 3.0 (Protein A HP 1 ml column).

For the in depth characterization of the soluble affinity ligands *via* Biolayer Interferometry (BLI), H9, M2 and M9 were chosen to be the most interesting molecules due to their pH-responsive behavior (H9), their magnesium-responsive binding in combination with high T_M_ (M9), or their combined magnesium and pH-responsive binding (M2). To this end, Daratumumab was immobilized onto anti-Human Fab-CH1 biosensors. The sensors were quenched in kinetics buffer and the association of the respective scFv was measured at different concentrations (Supplementary Figure S6). The dissociation was examined at pH 7.4, pH 5.0, and pH 4.5 respectively, as well as at pH 7.0 with 200, 400, and 600 mM MgCl_2_ added. H9 displayed high affinity binding with a *K*_D_ of 35.8 nM (Table 2). The dissociation rate increased with lower pH. A 21-, 44-, 161-fold enhanced dissociation was observed when placing the loaded tips in phosphate-citrate-buffer at pH 5.0, pH 4.5, and pH 4.0, respectively. Under similar conditions, M2 showed a 2.8, 5.4-, and 13-fold enhanced dissociation following the observations from the previous on-column experiments, where H9 was significantly more pH-responsive than M2. M2 and M9 exhibit medium affinity with *K*_D_ values in the triple digit nanomolar range of 504 and 290 nM, respectively. Interestingly, M2 and M9 did only display a slightly increased dissociation with increasing magnesium chloride concentrations (Table 3). We also checked for cross-reactivity of M2, M9, and H9 toward IgG4 antibodies. M2 and M9 did not show any binding signal toward IgG4, whereas H9 did bind IgG4 with comparable affinity as IgG1 (data not shown). Further cross-reactivity checks have not been performed since IgG1 and IgG4 are the most relevant isotypes in a therapeutic context (Irani et al., 2015).

**TABLE 2:** Kinetic data gathered *via* BLI for M2, M9, and H9.

	*K_D_ [nM]*	*K_D_ Error [nM]*	*k_on_ [M^–1^s^–1^]*	*k_on_ Error [M^–1^s^–1^]*	*k_dis_ [s^–1^]*	*k_dis_ Error [s^–1^]*
*M2*	504	±20.9	6.93 × 10^4^	±2.52 × 10^3^	3.50 × 10^–2^	±6.89 × 10^–4^
*M9*	290	±16.1	6.01 × 10^4^	±2.89 × 10^3^	1.74 × 10^–2^	±4.83 × 10^–4^
*H9*	35.9	±0.55	6.41 × 10^4^	±2.53 × 10^2^	2.30 × 10^–3^	±3.39 × 10^–5^

*Data was obtained on an Octet RED96 system (ForteBio, Pall Life Science) with FAB2G sensors. Fitting was performed with ForteBio Data Analysis 9.0.0.14.*

**TABLE 3:** Comparison of dissociation constants *k*_dis_ at pH 7.4, pH 5.0, pH 4.5, pH 4.0 and 200 mM MgCl_2_, 400 mM MgCl_2_, 600 mM MgCl_2_, respectively.

	*pH 7.4*		*pH 5.0*		*pH 4.5*		*pH 4.0*		*MgCl_2_ 200 mM*		*MgCl_2_ 400 mM*		*MgCl_2_ 600 mM*	
							
	*k*_dis_ [s^–1^]	*k*_dis_ Error [s^–1^]	*k*_dis_ [s^–1^]	*k*_dis_ Error [s^–1^]	*k*_dis_ [s^–1^]	*k*_dis_ Error [s^–1^]	*k*_dis_ [s^–1^]	*k*_dis_ Error [s^–1^]	*k*_dis_ [s^–1^]	*k*_dis_ Error [s^–1^]	*k*_dis_ [s^–1^]	*k*_dis_ Error [s^–1^]	*k*_dis_ [s^–1^]	*k*_dis_ Error [s^–1^]
*M2*	3.59 × 10^–2^	9.68 × 10^–4^	1.02 × 10^–1^	1.42 × 10^–3^	1.92 × 10^–1^	3.65 × 10^–3^	4.77 × 10^–1^	1.50 × 10^–2^	1.06 × 10^–1^	1.39 × 10^–3^	1.54 × 10^–1^	2.45 × 10^–3^	2.06 × 10^–1^	3.34 × 10^–3^
Enhancement [x-fold]*	1		2.8		5.4		13		3.0		4.3		5.7	
*M9*	2.75 × 10^–1^	1.78 × 10^–2^	n.d.	n.d.	n.d.	n.d.	n.d.	n.d.	3.29 × 10^–1^	2.35 × 10^–2^	4.10 × 10^–1^	3.27 × 10^–2^	4.67 × 10^–1^	4.59 × 10^–2^
Enhancement [x-fold]*	1								1.2		1.5		1.7	
*H9*	2.55 × 10^–3^	2.41 × 10^–3^	5.35 × 10^–2^	9.03 × 10^–4^	1.13 × 10^–1^	1.89 × 10^–3^	4.11 × 10 ^–1^	8.07 × 10^–3^	n.d.	n.d.	n.d.	n.d.	n.d.	n.d.
Enhancement [x-fold]*	1		21		44		161							

Calculations were performed with ForteBio Data Analysis 9.0.0.14. *Enhancement in comparison to the respective dissociation constant *k*_dis_ at pH 7.4 and 0 mM MgCl_2_ is shown.

## Discussion

In this work, we describe the fast and straightforward generation of chicken derived antibody fragments (scFvs) which can be used as ligands in affinity chromatography. By using yeast surface display and FACS it was possible to isolate chicken scFvs with pH-responsive and magnesium-responsive dissociation profiles which kept their responsive behavior upon immobilization on solid phase. Generation of scFv libraries in yeast *S. cerevisiae* is a well-established procedure (Feldhaus et al., 2003; Grzeschik et al., 2019) that routinely allows one to generate more than 10^9^ clones in a single working day. In this experiments, 5.2 × 10^9^ yeast transformants were obtained from harvested chicken B-cells, where more than 90% of the obtained clones contained a full length scFv encoding gene.

The screening for pH-responsive scFvs was performed according to previously reported screening campaigns for the isolation of pH-responsive antibody fragments (Könning et al., 2016; Bogen et al., 2019). In total, four sorting rounds were necessary to isolate scFvs with desired properties including only one negative sorting step to omit non-responsive binders. Likewise, the isolation of magnesium-responsive scFvs was performed within five sorting rounds including two consecutive negative selection rounds (Figure 1).

A small subset of only 10 single clones of each campaign was further analyzed, of which most displayed pH or magnesium-responsive binding, or both which indicates that this FACS-based shuttle-screening is a simple and robust procedure to enrich antibodies with prescribed binding characteristics.

Sequence analysis revealed seven unique pH-responsive and five unique magnesium-responsive scFv variants which could be allocated to three clusters each. No cluster was shared between both families, despite the fact that some of the magnesium-responsive binders also displayed pH-dependent binding. pH-dependent binding is often associated with a higher occurrence of histidine residues in the antibody complementarity determining regions since histidine is the only amino acid that is uncharged at neutral pH and becomes positively charged at acidic pH. The positive charge at lower pH can induce repulsive electrostatic interactions in cases where positive charges of the bound antigen are in close proximity to the antibody binding site (Murtaugh et al., 2011). We have not observed unusual strong enrichment of histidines in our described clones (data not shown). In addition, the pH-responsive behavior can also be caused by destabilizing the VH/VL interaction as well as the CDR loop arrangement weakening the antigen-binding. Specific framework changes to exploit ionisable groups in order to generate pH/calcium-responsive proteins are described in literature (Gulich et al., 2000; Kellmann et al., 2017; Kanje et al., 2018). Since we have no detailed knowledge of the exact binding epitopes it is currently unclear, which amino acids in the CDRs and the bound target protein epitope and which type of interaction account for the observed pH responsive binding. Aspartate and glutamate are typically involved in magnesium complexation, which could interfere with antigen binding (Dudev and Lim, 2007; Brylinski and Skolnick, 2011; Khrustalev et al., 2016). Since these residues are also amenable to protonation at acidic pH, and the microenvironment of the antigen-antibody complex may occasionally promote a shift to higher pK of these residues, it is tempting to speculate that these effects account for the observed pH-responsive binding of several obtained clones, that have exclusively been screened for reduced binding at elevated magnesium concentration. Notably, the pH- and magnesium-dependent binding is not due to denaturation of the scFv at acidic pH or high magnesium concentrations since melting temperatures were largely unchanged under these conditions (Table 1). Furthermore, it was possible to verify that the addition of MgCl_2_ can stabilize the scFv and therefore elevate the observed melting temperature (Supplementary Figure S7).

All scFvs could be expressed in *E. coli* SHuffle T7 Express cells with low to medium expression levels. No efforts were made yet to optimize expression yields, e.g., by codon optimization or using culture media for high density growth. No efforts were made to optimize the scFv loading density and target antibody binding capacity. Coupling onto NHS-agarose columns was performed with 5 mg protein resulting in coupling efficiencies of 67-96%. If a 1:1 interaction is assumed, the column with the least amount of coupled scFv (M3, 67%) should be capable of capturing 18.6 mg antibody which was considered sufficient to evaluate the capturing performance.

Protein A is the gold standard for antibody purification using mild elution conditions requiring a pH less than 4.0 for release of bound antibody. H1, H6, H7, and H9 did show efficient elution up to pH 4.0 with over 95% recovery which exceeds the recovery of Protein A at pH 4.0. H7 and H9 – both originated from the same cluster – appeared to be the most pH-responsive variants with close to quantitative elution at pH 4.5 (Figure 2B). The magnesium-responsive variants M2, M3, and M6 also showed more efficient elution than Protein A at pH 4.0. However, the most magnesium-responsive variants M8 and M9 did not show pH-responsive Fc binding. We found that combining pH-changes dependent on Mg^2+^-concentration can significantly increase recovery. Particularly for M2 we found that low MgCl_2_ concentrations will increase the recovery from 40% to 90% at ∼pH 5.0 (Figure 4). Also, the flow rate decrease from 3 ml/min to 1 ml/min lead to an increase of approx. 5% at pH 4.6 for H9 (Supplementary Figure S5) indicating that elution conditions might be further tuneable if desired.

In recent years, the Capture Select^TM^ strategy was introduced that relies on the isolation of camelid VHH domain that selectively bind various type of antibodies. These have also been developed for antibody affinity purification and elution at acidic pH or using elevated magnesium concentrations (Hermans et al., 2017). Little information is available in literature about the screening procedure, but multiple formats against different antibody formats have been developed upon lama immunization and subsequent phage display screening (Klooster et al., 2007; Detmers et al., 2010; Reinhart et al., 2012). Notably, in comparison to camelid derived VHHs that require a magnesium burst of 1 M MgCl_2_ in combination with 40% propylene glycol for elution, chicken scFvs isolated in this work showed elution at neutral pH with significantly reduced MgCl_2_ concentration (400 mM).

The observed *K*_D_ values for H9, M2 and M9 ranged from 40 nM to over 500 nM, well in the range of affinities sufficient for affinity chromatography as described by literature (Anspach, 2004; Mondal et al., 2006; Chen et al., 2014). Watanabe et al. (2009) engineered pH-responsive Protein G variants by rational design comprising similar *K*_D_ values. Their best variant showed an affinity decrease of 80-fold at 4.0 which is clearly exceeded by H9 with an affinity decrease of 161-fold. The findings for the magnesium-buffer dependency of target release were surprisingly not correlating to the on-column experiments. M9 displayed better recovery but less affinity decrease in the BLI experiments compared to M2. This indicates that the artificial conditions in the BLI setup do not correlate very well to the binding conditions of the scFv and the antigen on column.

Lastly, usability of chicken scFvs for affinity chromatography was underlined by the fact that no decrease in column capacity was observed during the testing period over up to 18 chromatography cycles. Additionally, no decrease in AUC was observed in a repeated cycle experiment were five consecutive chromatography runs were performed with H7 (Supplementary Figure S8) after all test runs.

In summary, in this work, we describe as straightforward procedure for the isolation of chicken antibody-based affinity ligands for affinity chromatography. We demonstrated that the immunization of chicken and the subsequent FACS selection process delivers a plethora of different ligands which can be individually optimized for chromatography applications. It is important to note that this generic strategy can also be applied to the purification of proteins with therapeutic relevance other than monoclonal antibodies.

## Data Availability Statement

The raw data supporting the conclusions of this article will be made available by the authors, without undue reservation, to any qualified researcher.

## Author Contributions

SH and HK conceived and designed the experiments, and wrote the manuscript. SH, AE, and AA performed the experiments. AE performed the experiments for the revision. SH, AE, OR, AS, and HK analyzed the data. OR, AS, SZ, and TH gave scientific advice. All authors contributed to the article and approved the submitted version.

## Conflict of Interest

TH, AS, OR, and SZ were employed by Merck KGaA. Merck KGaA has an interest in and develops resins for affinity chromatography. The remaining authors declare that the research was conducted in the absence of any commercial or financial relationships that could be construed as a potential conflict of interest.
