# Supplementary material for: A Generic Procedure for the Isolation of pH- and Magnesium-Responsive Chicken scFvs for Downstream Purification of Human Antibodies
Source: Front Bioeng Biotechnol. 2020 Jun 23;8:688. doi: 10.3389/fbioe.2020.00688 (PMC7324474; doi:10.3389/fbioe.2020.00688)
Supplement: Supplementary file 1 [file Table_1.DOCX]

Supplementary Material

# Supplementary Data

**Table S1.** Detailed staining strategy for all sorting rounds including the dilution of working solution for primary and secondary labeling agents.

|  | Surface Presentation | | TARGET BINDING | | |
| --- | --- | --- | --- | --- | --- |
| Sorting round | Primary labeling agent | Secondary labeling agent | Primary labeling agent | | Secondary labeling agent |
| 1. | anti-c-myc biotin antibody (1:30) | Streptavidin-PE (1:75) | 1 µM Fc-protein-dylight650 | - | |
| 2. | anti-c-myc biotin antibody (1:30) | Streptavidin-PE (1:75) | 1 µM Fc-protein-dylight650 | - | |
| 3. | anti-c-myc antibody (undil.) | anti-mouse IgG R-PE (1:30) | 1 µM Daratumumab | anti-Human IgG (Fab specific) –FITC (1:20) | |
| 4. pH | anti-c-myc antibody (undil.) | anti-mouse IgG R-PE (1:30) | 250 Pertuzumab | anti-Human IgG (Fab specific) –FITC (1:20) | |
| 5. pH | anti-c-myc biotin antibody (1:30) | Streptavidin-APC (1:75) | 150 nM Daratumumab | Goat F(ab')_2_ anti-Human Kappa-PE (1:80) | |
| 4. MgCl_2_ | anti-c-myc biotin antibody (1:30) | Streptavidin-PE (1:75) | 1 µM Cetuximab | anti-Human IgG (Fab specific) –FITC (1:20) | |
| 5. MgCl_2_ | anti-c-myc antibody (undil.) | anti-mouse IgG FITC (1:20) | 1 µM Cetuximab | Goat anti-Human IgG Fc Secondary Antibody PE (1:80) | |
| 6. MgCl_2_ | anti-c-myc biotin antibody (1:30) | Streptavidin-APC (1:75) | 150 nM Daratumumab | Goat F(ab')_2_ anti-Human Kappa-PE (1:80) | |

**Table S2**. Immuno-staining procedure of yeast cells before cell sorting. Cells were stained in a total volume of 20 µL per 1 × 10^7^ yeast cells. Every incubation step was followed by a wash step (W) using 1 mL PBS-B pH 7.4 (PBS + 0.1% (w/v) BSA) except for the last wash step which was performed twice to reduce background fluorescence. All staining and wash steps were performed at 4 °C.

| Incubation steps | | | | | | | | |
| --- | --- | --- | --- | --- | --- | --- | --- | --- |
| Sorting round | Incubation with target protein in PBS-B pH 7.4; 30 min; 4 °C | W | Negative selection step (either 5 min incubation in 1 mL phosphate citrate pH 5.0 or 30 s in Tris/HCl 50 mM pH 7.0, MgCl_2_ 2 M) | W | Primary labeling agents for surface detection and target binding in PBS-B pH 7.4; 15 min; 4 °C | W | Secondary labeling agents for surface detection and target binding in PBS-B pH 7.4; 15 min; 4 °C | W |
| Sorting campaign for the isolation of pH-responsive scFvs | | | | | | | | |
| 1. | **+** | **1x** |  | **1x** | **+** | **1x** | **+** | **2x** |
| 2. | **+** | **1x** |  | **1x** | **+** | **1x** | **+** | **2x** |
| 3. | **+** | **1x** |  | **1x** | **+** | **1x** | **+** | **2x** |
| 4. pH | **+** | **1x** | phosphate citrate pH 5.0 | **1x** | **+** | **1x** | **+** | **2x** |
| 5. pH | **+** | **1x** |  | **1x** | **+** | **1x** | **+** | **2x** |
| Sorting campaign for the isolation of MgCl_2_-responsive scFvs | | | | | | | | |
| 1. | **+** | **1x** |  | **1x** | **+** | **1x** | **+** | **2x** |
| 2. | **+** | **1x** |  | **1x** | **+** | **1x** | **+** | **2x** |
| 3. | **+** | **1x** |  | **1x** | **+** | **1x** | **+** | **2x** |
| 4. MgCl_2_ | **+** | **1x** | Tris/HCl 50 mM pH 7.0, MgCl_2_ 2 M | **1x** | **+** | **1x** | **+** | **2x** |
| 5. MgCl_2_ | **+** | **1x** | Tris/HCl 50 mM pH 7.0, MgCl_2_ 2 M | **1x** | **+** | **1x** | **+** | **2x** |
| 6. MgCl_2_ | **+** | **1x** |  | **1x** | **+** | **1x** | **+** | **2x** |

**Table S3.** Primers used for library generation and reformatting into an expression plasmid. Sequences are depicted in 5’ to 3’ orientation.

| *VH_gr_up* | GGTGGTGGTGGTTCTGGTGGTGGTGGTTCTGCTAGCGCCGTGACGTTGGACGAG |
| --- | --- |
| *VH_SOE_lo* | TCCGCCCCCCGACCCGCCGCCGCCTGAGCCGCCTCCCCCGGAGGAGACGATGACTTCGGT |
| *VL_SOE_up* | GGCGGCTCAGGCGGCGGCGGGTCGGGGGGCGGAGGGAGCGCGCTGACTCAGCCGTCCTCG |
| *VL_gr_lo* | CAAGTCCTCTTCAGAAATAAGCTTTTGTTCGGATCCTAGGACGGTCAGGGTTGTCCC |
| *scFv_chick_his_GG_up* | ATATATGGTCTCAATGGCTCACCACCACCACCACCACGGGTCTGCTAGCGCCGTGACGTTGGACGAGTCCG |
| *scFv_chick_SII_GG_*lo | ATATATGGTCTCATCATCATTTTTCGAACTGCGGGTGGCTCCAAGACCCTAGGACGGTCAGGGTTGTCCCGGCC |


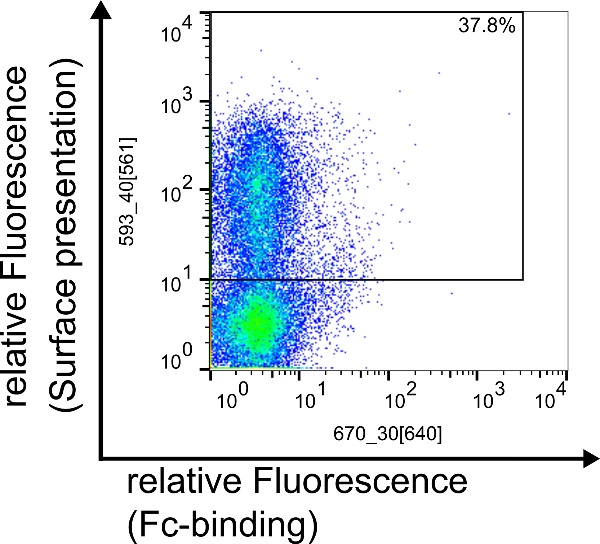


**Figure S1.** Depiction of 50,000 events of the initial, unsorted yeast library with target binding on the x-axis and surface presentation on the y-axis to determine the presentation levels.


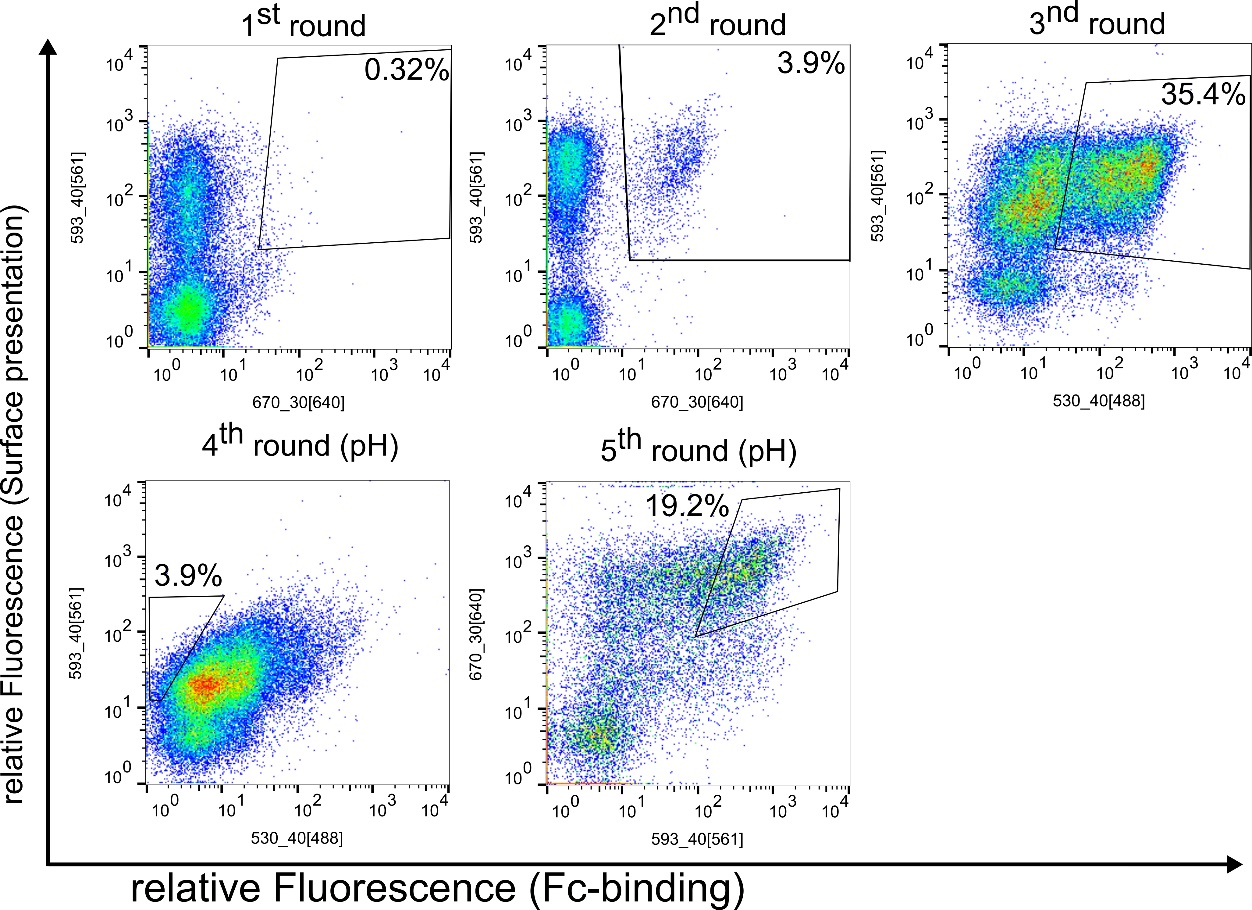


**Figure S2.** Summary of the sorting campaign for the isolation of pH-responsive chicken-scFvs. Each FACS plot shows 50,000 events with Fc-binding plotted on the x-axis and surface presentation on the y‑axis. Detailed staining strategy is summarized in Table S1. Enrichment of Fc-specific scFvs *via* YSD and FACS during the 1^st^, 2^nd^ and 3^rd^ sorting rounds. The 4^th^ sorting round was performed to deplete non-pH-responsive scFvs with an additional washing step with phosphate citrate buffer (Table S2). Last sorting round was conducted to isolate single clones positive for IgG binding at pH 7.4.


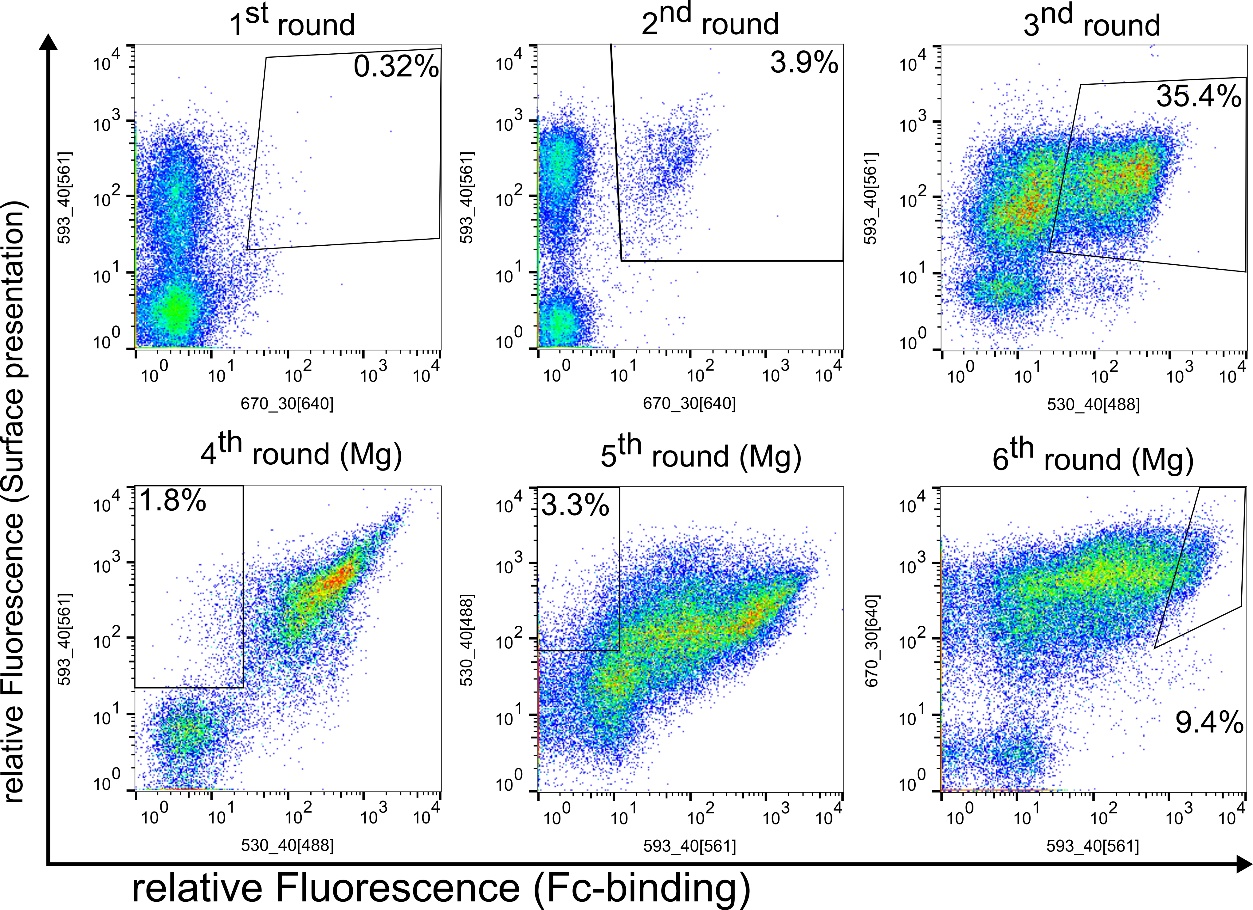


**Figure S3.** Summary of the sorting campaign for the isolation of magnesium-responsive chicken-scFvs. Each FACS plot shows 50,000 events with Fc-binding plotted on the x-axis and surface presentation on the y‑axis. Detailed staining strategy is summarized in Table S1. Enrichment of Fc-specific scFvs *via* YSD and FACS during the 1^st^, 2^nd^ and 3^rd^ sorting rounds. The 4^th^ and 5^th^ sorting rounds were performed to deplete non-magnesium-responsive scFvs with an additional washing step with MgCl_2_-containing buffer (Table S2). Last sorting round was conducted to isolate single clones positive for IgG binding at pH 7.4.

**A**

**B**


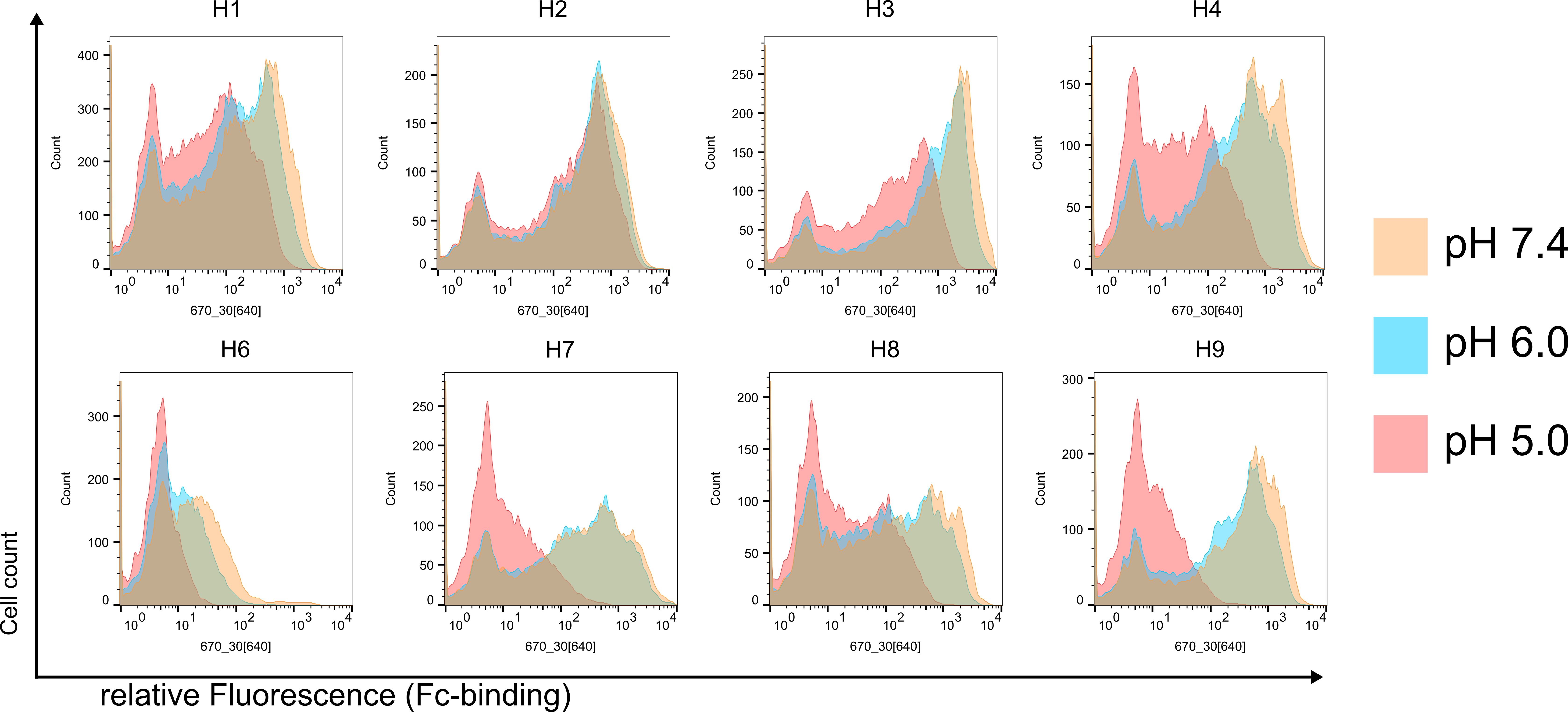


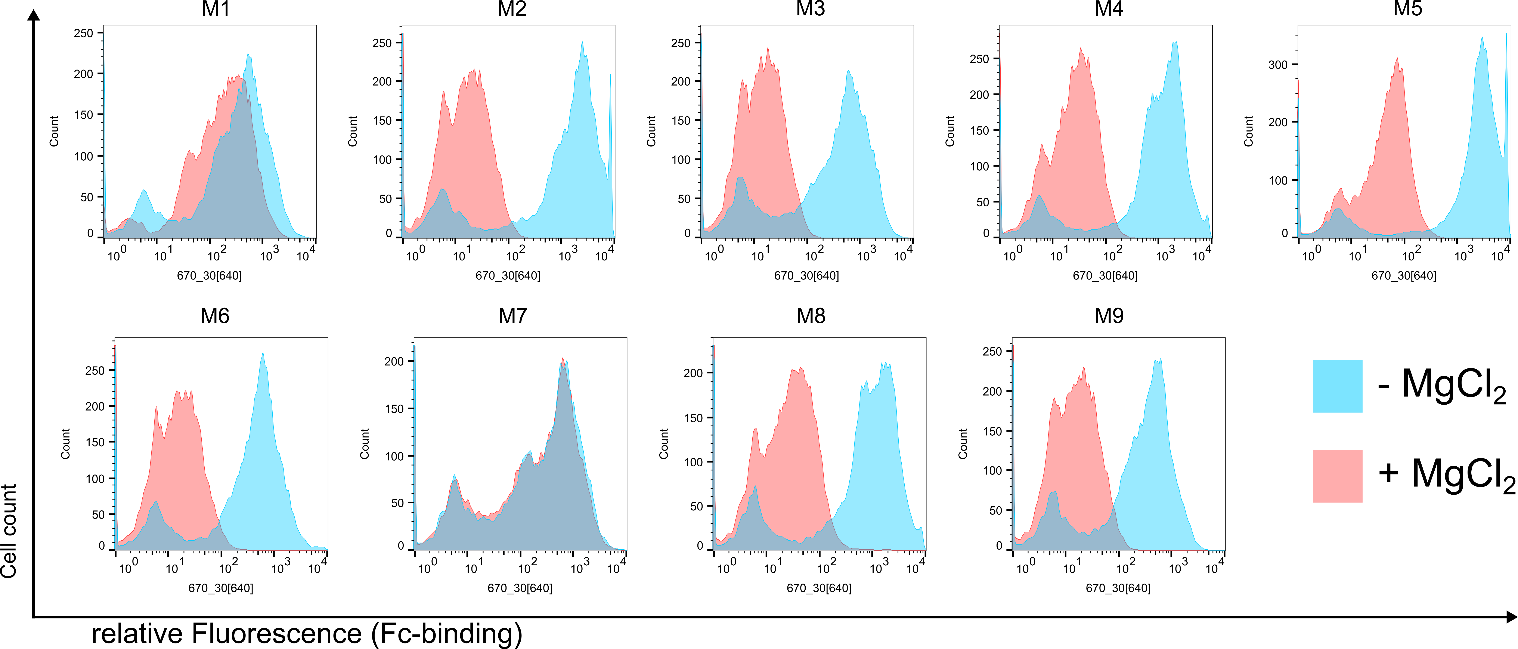


**Figure S4.** Summary of the single clone analysis by FACS. Each histogram contains 50,000 events with Fc-binding plotted on the x-axis and cell count on the y‑axis. Detailed staining strategy is summarized in Table S1, the staining procedure in Table S2. **(A)** Analysis of isolated putative pH‑responsive single clones. The washing step after target protein incubation was performed at pH 7.4 (orange), pH 6.0 (blue) or pH 5.0 (red), respectively. **(B)** Analysis of isolated putative magnesium‑responsive single clones. The washing step was either performed with non‑MgCl_2_‑containing Tris buffer (blue) or with Tris buffer containing 2 M MgCl_2_ (red).





**Figure S5.** Recovery for H9 at different flow rates. The recovery is depicted on the y-axis, the pH value on the x-axis. Each dot represents a single measurement. Red data points indicate a utilized flow rate of 1 mL/min while blue data points depict a flow rate of 3 mL/min. Vertical dashed lines indicate pH 4.0, pH 4.5 and pH 5.0, respectively, horizontal dashed lines indicate 90% recovery.


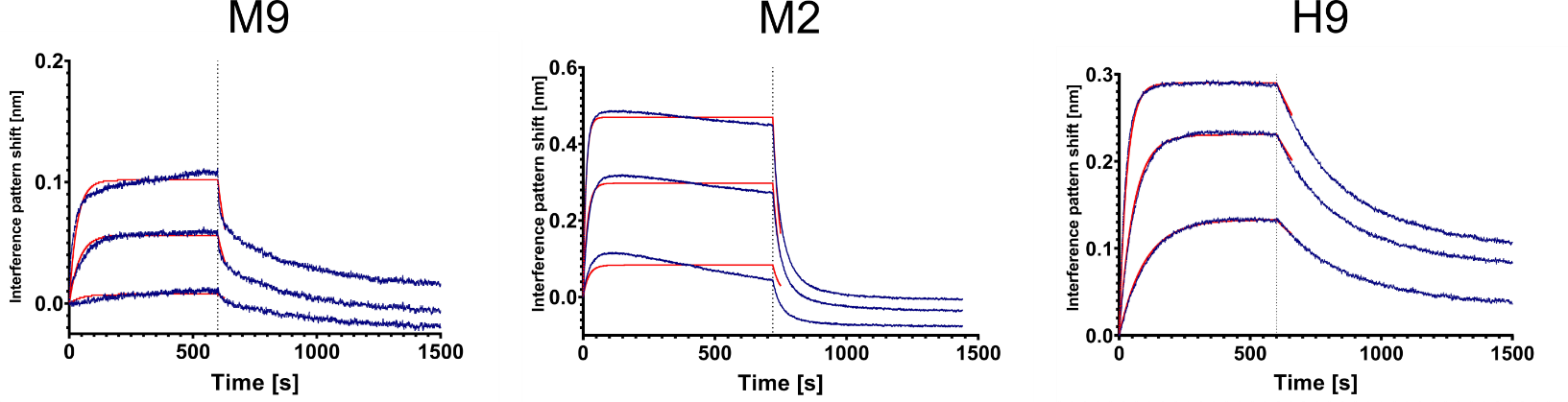


**Figure S6.** Binding kinetics of M9, M2 and H9 as determined via BLI on an Octet® RED96 system with the ForteBio data analysis software 9.0. Daratumumab was immobilized on FAB2G sensor tips and scFvs were subsequently associated at varying concentrations (M2: 720 s, M9/H9: 600 s). Dissociation was measured in PBS buffer at pH 7.4 for 600 s.





**Figure S7.** T_M_ measurements of scFv M9 via Nano DSF at different MgCl_2_ concentrations. Measurements were performed in singlets with a temperature gradient of 2°C/min.





**Figure S8.** Repetition experiments for pH-responsive scFv H7. Five chromatography runs were performed with identical conditions. For this chromatogram, the elution from 14-24 mL elution volume are depicted. Elution step 1 is performed at pH 4.52, the second elution step at pH 3.26 Each run is indicated by a unique color, indicated on the right. Individual runs are nudged by 50 mAU and 0.3 mL.


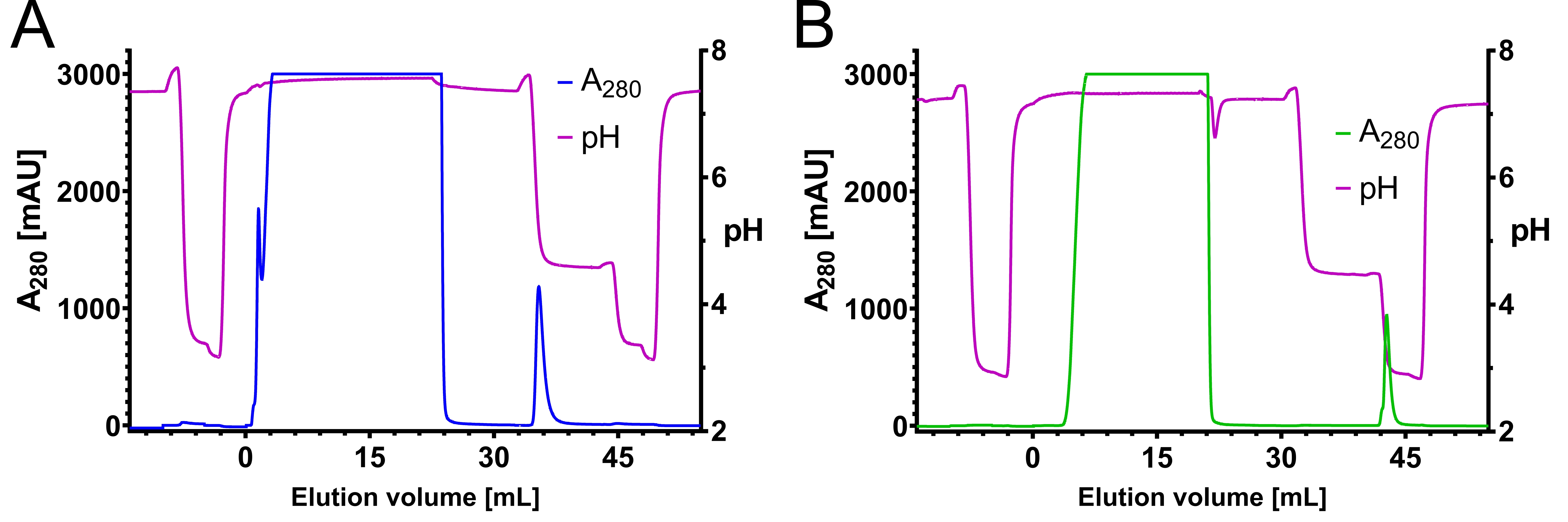


**Figure S9.** Trastuzumab purification from trastuzumab producing Expi293F cell culture supernatant. (A) Chromatogram of the purification of trastuzumab from 15 mL Expi293 supernatant. Absorbance at 280 nm is depicted on the left y-axis (blue), pH is shown on the right y-axis (violet). The elution volume is depicted on the x-axis. Elution was performed in two steps with pH 4.6 and pH 3.0, repectively. (B) Chromatogram of the purification of trastuzumab from 15 mL Expi293 supernatant with a Protein A HP column manufactured by GE Healthcare. Absorbance at 280 nm is depicted on the left y-axis (green), pH is shown on the right y-axis (violet). The elution volume is depicted on the x-axis. Elution was performed in two steps with pH 4.6 and pH 3.0, repectively.
